# An AI-Designed Antibody-Engineered Probiotic Therapy Targeting Urease to Combat *Helicobacter pylori* Infection in Mice

**DOI:** 10.3390/microorganisms13092043

**Published:** 2025-09-01

**Authors:** Feiliang Zhong, Xintong Liu, Xuefang Wang, Mengyu Hou, Le Guo, Xuegang Luo

**Affiliations:** 1 Key Laboratory of Industrial Fermentation Microbiology of the Ministry of Education, College of Biotechnology, Tianjin University of Science and Technology, Tianjin 300457, China; flzhong91@tust.edu.cn (F.Z.); liuxintong0514@163.com (X.L.); wxf21116@163.com (X.W.); 17733599165@163.com (M.H.); 2 Ningxia Key Laboratory of Clinical Pathogenic Microorganisms, School of Laboratory Medicine, Ningxia Medical University, Yinchuan 750004, China; guoletian1982@163.com

**Keywords:** *Helicobacter pylori*, AI-driven antibody design, Ab single-domain antibodies, *Escherichia coli* Nissle 1917 engineered probiotics, flora homeostasis

## Abstract

*Helicobacter pylori* (Hp), a Class I carcinogen infecting over 50% of the global population, is increasingly resistant to conventional antibiotics. This study presents an AI-engineered probiotic strategy targeting urease, a key Hp virulence factor. A humanized single-domain antibody (UreBAb), previously identified and selected in our laboratory, was synthesized commercially and modeled using AlphaFold2, with structural validation conducted via SAVES 6.0. Molecular docking (PyMOL/ClusPro2) and binding energy analysis (InterProSurf) identified critical urease-active residues: K40, P41, K43, E82, F84, T86, K104, I107, K108, and R109. Machine learning-guided optimization using mCSA-AB, I-Mutant, and FoldX prioritized four mutational hotspots (K43, E82, I107, R109), leading to the generation of nine antibody variants. Among them, the I107W mutant exhibited the highest activity, achieving 65.6% urease inhibition—a 24.95% improvement over the wild-type antibody (*p* < 0.001). Engineered *Escherichia coli* Nissle 1917 (EcN) expressing the I107W antibody significantly reduced gastric HP colonization by 4.42 log10 CFU in the treatment group and 3.30 log10 CFU in the prevention group (*p* < 0.001 and *p* < 0.05, respectively), while also suppressing pro-inflammatory cytokine levels. Histopathological (H&E) analysis confirmed that the I107W antibody group showed significantly enhanced mucosal repair compared to wild-type probiotic-treated mice. Notably, 16S rRNA sequencing revealed that intestinal microbiota diversity and the abundance of core microbial species remained stable across different ethnic backgrounds. By integrating AI-guided antibody engineering with targeted probiotic delivery, this platform provides a transformative and microbiota-friendly strategy to combat antibiotic-resistant Hp infections.

## 1. Introduction

*Helicobacter pylori* (Hp), a Gram-negative, microaerophilic bacterium, colonizes the human gastric mucosa through unique adaptations including urease-mediated acid resistance, infecting over 50% of the global population [1]. Classified as a Group 1 carcinogen by the International Agency for Research on Cancer (IARC), Hp is etiologically linked to gastric cancer and extra-gastrointestinal pathologies such as cardiovascular disorders, dermatological conditions, and adverse pregnancy outcomes [2,3,4]. While antibiotic combination therapies remain first-line treatments, escalating resistance—exemplified by clarithromycin resistance rates rising from 15% to 42% within a decade—has driven global eradication efficacy below 55% [5,6]. In China, where antibiotic resistance trends mirror global patterns, conventional regimens increasingly fail to achieve therapeutic targets, underscoring the urgent need for innovative strategies.

Against this backdrop, breakthroughs in artificial intelligence technology offer a fresh perspective on anti-Hp treatment. The advantage of AI-assisted design is that it reduces a lot of economic costs and is efficient [7]. AI-assisted methods are widely regarded as key tools for small-molecule drug discovery, and they help optimize target affinity, minimize non-target effects, and optimize pharmacokinetic properties [8]. Accurate prediction of antibody structure is an important issue in protein research. Although one must initiate antibody design with a pre-existing, laboratory-designed antibody, AI-assisted design reduces the cost of producing optimized variants of the original antibody by efficiently generating candidates in silico, which can then be recombinantly produced and laboratory tested. When we are faced with situations where the binding between antibodies and antigens is unknown, protein–protein docking methods can be used to predict complex antibody structures [9,10]. Although there are still some limitations when modeling structures using homology of antibodies or antigens, this is a promising approach. The structure of the antibody–antigen complex that has been experimentally determined or predicted can be further utilized, and computational methods can be employed to predict mutations that may enhance binding affinity, specificity, or other properties such as solubility [11,12].

Concurrently, probiotic delivery systems demonstrate multifaceted anti-Hp mechanisms, including competitive exclusion, immunomodulation, and mucosal barrier enhancement [13,14,15,16]. Clinical studies reveal that probiotic adjuvants elevate eradication rates, reduce antibiotic-associated diarrhea, and improve treatment compliance across diverse strains and dosing regimens [17]. Despite proven efficacy against pathogens like *Pseudomonas aeruginosa* and metabolic disorders, engineered probiotics remain underexplored for Hp eradication a critical gap given Hp’s gastric tropism and emerging extra-gastric associations, including intestinal colonization in ulcerative colitis patients [18].

An infection in the stomach, as the main colonization site of Hp, can cause various diseases such as gastritis, peptic ulcers, and even gastric cancer [19]. Furthermore, studies have found that Hp is also present in the intestinal mucosa of patients with ulcerative colitis, suggesting that Hp may be present [20] in multiple locations outside the stomach. Meanwhile, engineered probiotics have been validated in animal models for treating Pseudomonas aeruginosa intestinal infections, hepatic encephalopathy, hyperuricemia and other diseases, but there are few [21,22] reports on the use of engineered probiotics to treat Hp. In addition, AI-assisted antibody design provides new ideas for experiments. Deep learning-based models can better predict the interaction between antibodies and Hp proteins, thereby optimizing antibody affinity and specificity [23].

This study integrates AI-driven antibody optimization with probiotic engineering to target Hp. The urease structure, modeled using AlphaFold2, was validated with SAVES6.0, and key active-site residues were identified through molecular docking via ClusPro2 and InterProSurf. Computational screening (mCSA-AB, I-Mutant, FoldX) generated nine antibody variants, with the optimal mutant (I107W) selected for expression in *E. coli* Nissle 1917 (EcN). Protein expression and anti-urease activity were confirmed using SDS-PAGE and phenolphthalein assays. The therapeutic efficacy of the engineered I107W Ab probiotic against Hp colonization was further validated in a mouse model.

## 2. Materials and Methods

### 2.1. Molecular Dynamics Analysis and Evolutionary Design of Single-Domain Antibodies

The anti-Hp monoclonal antibody used in this study originated from prior work in our laboratory. Intermolecular interactions between various antibodies and the Hp urease subunit B (UreB) were analyzed using PyMOL, I-TASSER, and ClusPro2, leading to the identification of the UreBAb sequence, a fully humanized single-domain variable light chain (VL) antibody targeting UreB (113 amino acids), hereafter referred to as “Ab” [24]. The antibody gene was synthesized by Suzhou Genewiz Biotechnology Co., Ltd. (Suzhou, China) and cloned into the pUC57 vector. The target fragment was subsequently amplified via PCR. Structural modeling was performed by inputting the antibody sequence into AlphaFold2 (https://alphafoldserver.com/ (accessed on 2 November 2022)) and selecting the top-ranked predicted PDB file based on confidence scores. Model validation was conducted using SAVES 6.0 (https://saves.mbi.ucla.edu/ (accessed on 4 November 2022)), which included Ramachandran plot analysis and ERRAT evaluation. The Ramachandran plot showed that over 90% of residues were located in energetically favorable regions, indicating proper backbone geometry [25]. ERRAT analysis quantified structural reliability, where yellow regions represented residues exceeding the 95% confidence rejection threshold—a feature present in <5% of high-quality protein structures, while red regions indicated critical errors at the 99% confidence level [26]. These combined metrics confirmed the model’s compliance with empirical protein structural standards and its suitability for subsequent docking analyses.

Prior to protein docking, both antigen and antibody structures were pre-processed to improve docking accuracy. Using PyMOL (https://pymol.org/2/ (accessed on 4 November 2022)), water molecules, metal ions, and other interfering factors were removed. The urease structure (PDB ID: 1E9Z) was downloaded from the Protein Data Bank (https://www.rcsb.org/). The processed antigen and antibody structures were then uploaded to the ClusPro protein-protein docking server (https://cluspro.org/help.php (accessed on 4 November 2022)), selecting the antibody docking protocol.

Following successful docking complex construction, active-site prediction and candidate mutation site analysis were performed. The complex structure was uploaded to the InterProSurf server (https://curie.utmb.edu/pdbcomplex.html (accessed on 10 November 2022)) to analyze surface residues and interfacial characteristics. This tool predicts surface amino acids involved in protein–protein interactions, providing interfacial residue lists, interface areas, and surface area changes upon complex formation. PyMOL was further used to visualize inter-chain interactions within a 4.0 A range, allowing identification of key residues critical for binding affinity.

Once mutation sites were determined, three computational tools—mCSM-AB, FoldX, and I-Mutant—were employed to evaluate the antibody’s evolutionary potential. mCSM-AB predicted changes in Gibbs free energy (∆∆G = ΔGwt − ΔGmt), while FoldX used the following command for mutational scanning: FoldX –command = PositionScan –pdb = A.pdb –positions = position/chain/a. Here, a denotes scanning against all 20 common amino acids. Other options included d: 24 residues (20 standard + phosphorylated Tyr, Thr, Ser, and hydroxyproline); h: a hydrophobic residue set {“GLY”, “ALA”, “LEU”, “VAL”, “ILE”, “THR”, “SER”, “CYS”, “MET”, “LYS”, “TRP”, “TYR”, “PHE”, “HIS”}; c: a charged residue set {“ARG”, “LYS”, “GLU”, “ASP”, “HIS”}; and p: polar residues {“ARG”, “LYS”, “GLU”, “ASP”, “HIS”, “TRP”, “TYR”, “THR”, “SER”, “GLN”, “ASN”}. FoldX calculates free energy changes as ∆∆G = ∆Gmt − ∆Gwt, providing insight into the effects of mutations on antibody stability and binding affinity.

### 2.2. Single-Point Mutation and Vector Construction

To validate the AI-predicted structural models, plasmid DNA was extracted from the laboratory-constructed pET28a-Ab vector and used as the template for reverse PCR with specifically designed single-point mutation primers. Mutation efficiency was assessed via agarose gel electrophoresis. Verified mutant plasmids were transformed into *E. coli* BL21 competent cells. Overnight seed cultures were prepared and inoculated into 50 mL LB medium at a 1.5% inoculation ratio. Cultures were grown at 37 °C and 220 rpm until reaching an OD_600_ of approximately 0.7, at which point IPTG was added to a final concentration of 1 mmol/L to induce protein expression. Expression was continued under optimized conditions (25 °C, 200 rpm) for 20 h. Cells were harvested by centrifugation and lysed by ultrasonic disruption to obtain soluble protein fractions. To functionally validate the expressed mutant proteins, Hp strains cultured for 5–7 days were used to prepare crude pathogen extracts. The engineered bacterial lysates containing mutant antibodies were mixed with Hp crude extracts at a 1:1 ratio in phosphate buffer (pH 6.8). After 3 h of incubation with phenol red as a pH indicator, urease enzymatic activity was quantified by measuring absorbance at 550 nm using a microplate reader.

Separately, the PTM151 expression vector was subjected to double restriction digestion using SpeI and SgrAI. The linearized vector was ligated with the target gene fragment via homologous recombination. The recombinant construct was transformed into *E. coli* DH5α competent cells, and positive clones were selected for plasmid validation. Confirmed recombinant plasmids were then electroporated into EcN for heterologous expression. To verify expression of the target protein, Western blotting was performed using an anti-6xHis primary antibody (Proteintech), with GroEL (Abcam) as the internal control.

### 2.3. Molecular Dynamics Simulation

After docking, the complex was subjected to molecular dynamics simulation using the GROMACS 2023.5 software. The all-atom oplsaa force field was used for its topology generation. The complex was placed in a cubic box, with the distance between each atom and the edge of the box set to 1 nm, and solvated using the spc three-point water model. An appropriate number of sodium ions or chloride ions were added to ensure that the charge in the system was zero. Energy minimization and system pre-equilibration were carried out. After the system reached equilibrium, a 100 ns molecular dynamics simulation was performed at 310 K. After the simulation ended, periodic boundary condition processing was performed, and the trajectory was analyzed, including RMSD, RMSF, and the radius of gyration (Rg).

### 2.4. Animal Models

Five-week-old female BALB/c mice (purchased from Spafur) were used to evaluate the oral efficacy of the engineered probiotics. Prior to experimentation, mice were acclimated for seven days under controlled environmental conditions: temperature 22 ± 1 °C, relative humidity 50 ± 5%, a 12/12 h light/dark cycle, and 15 air changes per hour. Food and water were provided ad libitum. All animals were individually marked for identification prior to model establishment. To neutralize gastric acid, 1 mL of 0.2 mol/L NaHCO_3_ solution was administered orally. Meanwhile, a fresh suspension of Hp (5 × 10^10^ CFU/mL) was prepared and injected intraperitoneally at 0.1 mL per dose, once daily, for seven consecutive days. The Hp strain (ATCC43504) used in this study was kindly provided by Professor Le Guo of Ningxia Medical University. For the prevention group, modeling was initiated during the final week of the experiment. Detailed experimental protocols and treatment timelines are provided in Table 1. Multiple groups were established to assess therapeutic efficacy and potential synergistic effects: Negative Control (NC): received no treatment intervention. Model Control (Hp only): received Hp infection without therapeutic intervention. Positive Drug Control (Drug): treated with standard anti-Hp medication. Non-antibody Probiotic (Probiotic): administered EcN lacking antibody expression. Antibody-Engineered Probiotic (Ab Probiotic): received EcN engineered to express specific antibodies. Combination Therapy (Drug/Ab Probiotic): received both standard medication and antibody-engineered probiotics. Prophylactic Group (Prophylactic): received engineered probiotics to evaluate their preventive potential. Experimental grouping and administration schedules are summarized in Table 1.

### 2.5. Detection of Hp Colonization in the Stomach

Following dissection, the stomach tissues of the mice were excised and opened along the greater curvature to remove luminal contents. The cleaned gastric tissues were then homogenized using a tissue homogenizer to obtain uniform homogenates. One portion of the homogenate was plated onto Hp-selective solid medium containing appropriate additives. Plates were pre-inverted and incubated anaerobically at 37 °C for 3 to 5 days. Bacterial colonization was assessed by counting visible colonies. Another portion of the homogenate was incubated with phenol red indicator for over 3 h. Urease activity, indicative of Hp presence, was quantified by measuring absorbance at 550 nm using a microplate reader.

### 2.6. ELISA Detection of Inflammatory Cytokines

Homogenized gastric tissues from mice were centrifuged at 12,000 rpm for 10 min at 4 °C to collect the supernatant for cytokine analysis. ELISA kits were equilibrated to room temperature for 30 min prior to use. Standard and sample wells were prepared according to the manufacturer’s instructions. Appropriate volumes of standards and test samples were added to the designated wells, followed by the addition of HRP-conjugated detection antibodies. The plates were then incubated at 37 °C for 60 min. After washing, chromogenic substrates A and B were added sequentially, and the plates were incubated in the dark at 37 °C for 15 min. The enzymatic reaction was terminated with a stop solution, and absorbance at 450 nm was measured immediately using a microplate reader. This procedure enabled the quantitative detection of inflammatory cytokines including TNF-α, IFN-γ, and IL-4 in gastric tissue lysates.

### 2.7. 16S rDNA High-Throughput Sequencing of Gut Microbiota

Approximately 2 mL of cecal contents was collected from each mouse, flash-frozen in liquid nitrogen, and stored at −80 °C. Samples were then sent to the Bimake platform for 16S rRNA gene sequencing. Operational Taxonomic Unit (OTU) representative sequences were classified taxonomically using the Greengenes database to generate OTU counts and relative abundance curves (RACs) at various taxonomic levels for each sample. Species richness was assessed using the Chao1 and Observed Species indices, while species diversity was evaluated with the Shannon and Simpson indices. Together with rarefaction curves and species coverage data, these metrics provided a comprehensive analysis of alpha diversity within the microbial communities. To assess between-group variation, beta diversity analysis was performed, including non-metric multidimensional scaling (NMDS) to visualize differences in microbial community structures across samples. Based on OTU annotations, the microbial composition at both the phylum and genus levels was profiled and visualized through distribution maps.

### 2.8. Statistical Analysis

All data were analyzed using GraphPad Prism 8.0 software. One-way analysis of variance (ANOVA) was performed to assess statistical differences among groups. A *p*-value < 0.05 was considered statistically significant. Significance levels are indicated as follows: ns (not significant, *p* > 0.05), * *p* < 0.05, ** *p* < 0.01, *** *p* < 0.001. Data are presented as mean ± standard deviation (SD).

## 3. Results

### 3.1. Identification of Affinity-Enhancing Sites and Mutant Screening via Structural Prediction and Computational Optimization

**Figure 1:**
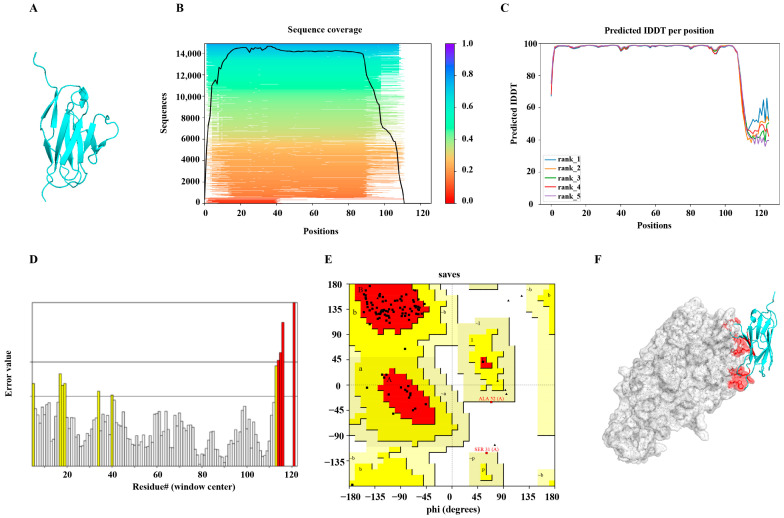
Structural modeling and docking analysis of the AI-designed single-domain antibody targeting urease. (**A**) Three-dimensional structure of the single-domain antibody predicted by AlphaFold2 homology modeling. (**B**,**C**) IDDT (inter-residue distance difference test) scores were used to evaluate prediction confidence, ranging from 0 to 100, with higher values indicating greater structural reliability. (**D**) ERRAT analysis chart showing model reliability. Yellow regions indicate segments exceeding the 95% confidence rejection threshold—commonly observed in ~5% of high-quality protein models—while red regions denote segments rejected at the 99% confidence level. (**E**) Ramachandran plot evaluating backbone dihedral angles (ψ and φ) to determine whether residues fall within energetically favorable regions. The red-colored areas represent the most favoured regions (A,B,L), the yellow-colored areas represent the additional allowed regions (a,b,l,p), and the light-colored areas represent the generously allowed regions (~a,~b,~l,~p). (**F**) Molecular docking simulation results. The urease structure is shown in gray, the antibody in blue, and predicted binding sites in red.

To enhance the antigen-binding activity of the original antibody, an AI-assisted optimization workflow was implemented, integrating homology modeling using AlphaFold2 for single-domain antibody structure prediction with three computational design refinement tools: mCSM-AB, I-Mutant, and FoldX (Figure 1A). The IDDT (inter-residue distance difference test) score was used to evaluate structural prediction confidence. Regions with IDDT > 80 exhibited high prediction reliability and were considered suitable targets for mutagenesis, while regions with IDDT < 40 (residues 100–120) showed low structural confidence and were excluded due to the potential risk of inducing unfavorable conformational changes (Figure 1B,C). To further evaluate the structural rationality of the predicted model, multiple antibody conformations were analyzed in detail (Figure 1D,E). Regions exhibiting high error values were flagged as structurally unstable and potentially mutation-sensitive, where even minor substitutions might impact protein stability or function. Overall, the model displayed a low error rate, indicating high structural reliability. The single-domain antibody comprised 113 amino acids, including 14 glycine and proline residues. Ramachandran plot analysis showed that 76.4% of residues were located in the most favored regions and 14.5% in additionally allowed regions, supporting the model’s structural validity. ERRAT analysis further confirmed that the final optimized model exhibited significantly improved quality compared to the initial version. The final docking configuration was visualized using PyMOL (v. 3.1) software (Figure 1F).

Structural analysis using PyMOL and InterProSurf identified ten key interaction residues within the antibody–antigen interface: K40, P41, K43, E82, F84, T86, K104, I107, K108, and R109. These residues were selected as candidate sites for targeted mutagenesis. A comprehensive screening workflow was applied: each of the 10 residues underwent exhaustive single-point substitution with 19 alternative amino acids. Changes in binding affinity and structural stability (ΔΔG values) were predicted using three computational tools—mCSM-AB, I-Mutant, and FoldX—with detailed results provided in Supplementary Tables S1–S4. In general, ΔΔG > 0 (in mCSM-AB) indicates enhanced complex stability and binding affinity, whereas ΔΔG < 0 suggests destabilization. FoldX and I-Mutant uses the opposite convention: ΔΔG < 0 denotes increased stability, and ΔΔG > 0 indicates destabilization. Cross-validation among the three algorithms consistently identified four hotspot residues—K43, E82, I107, and R109—as candidates for affinity enhancement through single-point mutations. Based on these findings, nine mutants were prioritized: K43L, K43T, K43M, E82W, I107P, I107W, I107F, R109P, and R109N (Table 2). Thermodynamic analysis revealed a favorable enthalpy–entropy profile for the I107W mutant. Specifically, mCSM-AB predicted a ΔΔG of +0.178 kcal/mol, indicating enhanced binding affinity, while FoldX predicted a ΔΔG of −0.103 kcal/mol, consistent with improved structural stability. These changes are attributed to enhanced hydrophobic interactions and potential hydrogen bonding contributions from the tryptophan side chain. Additionally, I-Mutant analysis suggested reduced entropic penalties (−1.78 kcal/mol), likely resulting from decreased conformational flexibility imparted by the rigid indole ring of tryptophan.

### 3.2. Engineering E. coli Nissle 1917 for High-Activity Expression of the Single-Domain Antibody Mutant (Ab-I107W)

**Figure 2:**
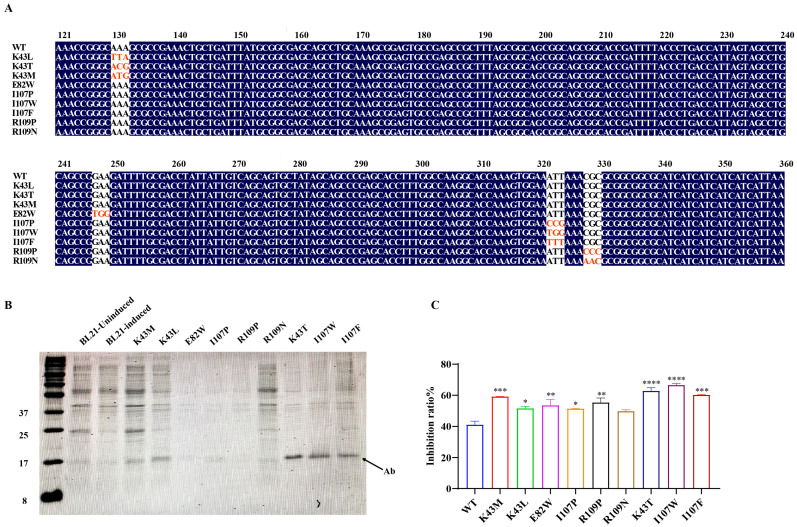
Verification, expression, and functional assessment of wild-type and mutant UreBAb antibodies targeting urease. (**A**) DNA sequencing confirmation of site-directed mutagenesis for the nine antibody variants. Red represents the base after mutation. (**B**) SDS-PAGE analysis of recombinant wild-type UreBAb and its nine mutant forms, confirming expected molecular weights. (**C**) Inhibition of urease activity by wild-type (WT) and mutant antibodies at 37 °C. Data are presented as mean ± SD. Statistical significance was determined relative to the WT: * *p* < 0.05, ** *p* < 0.01, *** *p* < 0.001, **** *p* < 0.0001.

Experimental validation of the computational predictions was carried out through site-directed mutagenesis at the identified hotspot residues, followed by heterologous expression of the resulting antibody variants in *E. coli* BL21 (Figure 2A). SDS-PAGE analysis confirmed the successful expression of all nine mutant proteins, with observed molecular weights (~12.5 kDa) consistent with theoretical values (Figure 2B). Notably, mutants K43T, I107W, and I107F demonstrated higher expression levels than other variants, surpassing even the parental wild-type antibody.

**Figure 3:**
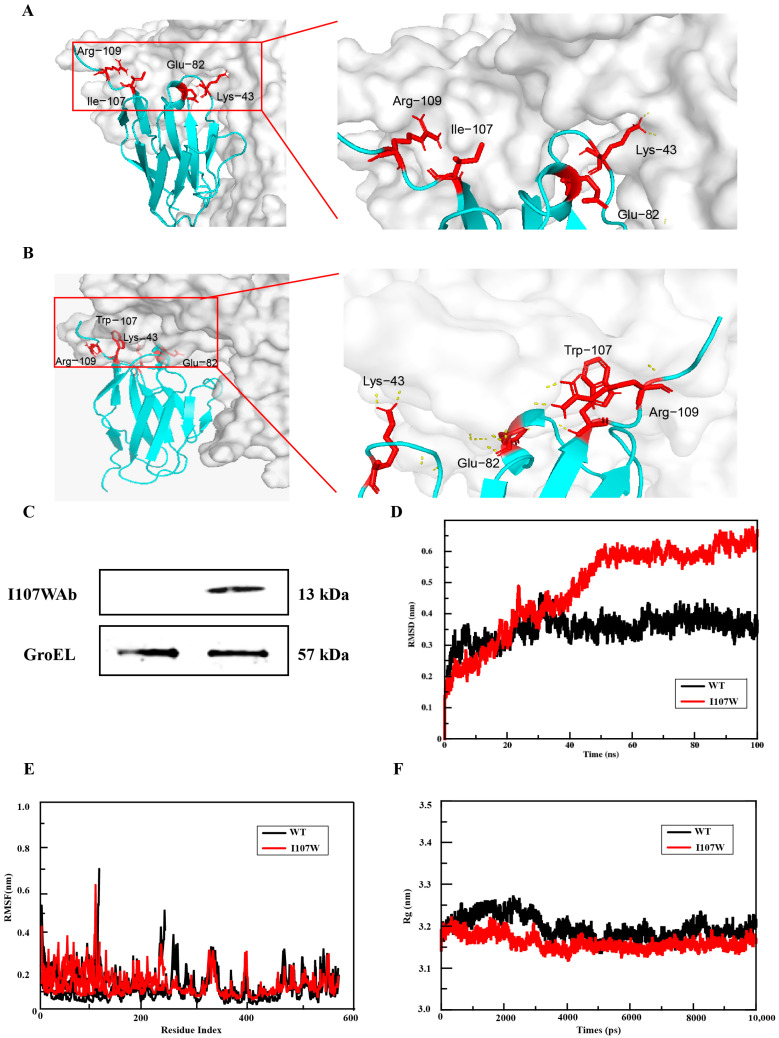
Docking simulations demonstrate enhanced binding of the I107W antibody mutant and confirm successful vector construction. (**A**) Molecular docking simulation of the wild-type antibody prior to mutation. The predicted binding site is highlighted in red. No hydrogen bonds were observed between residue 107 of the antibody and residue Y541 of urease. (**B**) Post-mutation docking analysis of the I107W variant. Hydrogen bonds were formed between residues 107, 43, 82, and 109 of the antibody and the urease, indicating enhanced binding interactions. (**C**) Western blot analysis confirming expression of the I107W mutant antibody in EcN. Left lane: empty PTM151 vector (negative control); right lane: PTM151-I107WAb construct. (**D**) RMSD plots of WT (black) and I107W mutant (red) over 100 ns molecular dynamics simulation. RMSD values reflect the overall structural deviation of the protein from its initial conformation. (**E**) RMSF profiles of WT (black) and I107W mutant (red) across residue indices. RMSF values represent the flexibility of individual residues. (**F**) Rg plots of WT (black) and I107W mutant (red) over molecular dynamics simulation (time in ps). Rg reflects the overall compactness of the protein.

Functional characterization of the mutants revealed enhanced antigen-binding affinity and urease-inhibitory activity across all variants, with the I107W substitution (isoleucine → tryptophan at position 107) exhibiting the most significant enhancement (Figure 3A,B). Molecular docking simulations of the wild-type antibody showed a lack of hydrogen bond formation between the antibody and urease, indicating weak or absent binding. In contrast, post-mutation simulations demonstrated new hydrogen bond interactions at the binding interface, confirming improved structural compatibility. The I107W mutant exhibited a 24.95% increase in binding activity (Figure 2C), suggesting that mutation-induced conformational stabilization may reduce proteolytic susceptibility and enhance functional integrity [22]. AlphaFold2-based structural modeling and ClusPro2 docking analyses further indicated that the I107W mutation promoted hydrophobic interactions and π–π stacking within the binding pocket, contributing to both the 24.95% increase in binding affinity and a 65.6% reduction in urease enzymatic activity. Additionally, electrostatic stabilization was observed at the antibody–urease interface. Specifically, a charge pair formed between K43 and E82 helped stabilize the complex, as molecular docking simulations revealed an 18.7 kJ/mol reduction in electrostatic potential difference following the mutation. Based on these findings, the I107W mutant was selected for probiotic system integration. The recombinant PTM151-I107WAb expression vector was successfully transformed and expressed in EcN, as confirmed by Western blotting using an anti-His antibody, with GroEL serving as the internal control (Figure 3C).

### 3.3. Molecular Dynamics Simulation Results of Mutants

Subsequently, molecular dynamics simulations were performed on the final selected mutant. The RMSD values were used to evaluate the binding stability of the ligand–receptor complex. The mean RMSD values of the complex within 100 ns of the molecular dynamics simulation runtime were calculated to compare the differences in binding stability between the nanobody and urease. The overall RMSD value of the I107W mutant was higher than that of the wild type (Figure 3D). It rose rapidly in the early stage and then became relatively stable, indicating good conformational stability.

The RMSF values were used to analyze the fluctuation of the ligand–receptor complex during the simulation. The vertical axis represents the RMSF value, and a larger value indicates more intense dynamic fluctuation of the corresponding residue. The overall fluctuation of the wild-type complex had high and low peaks, and the RMSF peaks in certain regions (such as those with low residue indices) were high, indicating that the residues at these positions in the wild type had high flexibility and active movement; the fluctuation in most regions was relatively stable, reflecting regular dynamics (Figure 3E). The residue fluctuation of the I107W mutant was generally similar to that of the wild type, but the peaks in some high-fluctuation regions were lower, and the fluctuation amplitude became gentler.

The radius of gyration was used to measure the overall compactness of the protein during the simulation. The change in the radius of gyration of the mutant (I107W) was similar to that of the wild type, but the overall value was slightly lower (closer to 3.1–3.2 nm) with a relatively smaller fluctuation amplitude, indicating that the mutation made the overall conformation of the molecule more compact (Figure 3F).

### 3.4. Engineered Probiotics Suppress Hp Colonization In Vivo

**Figure 4:**
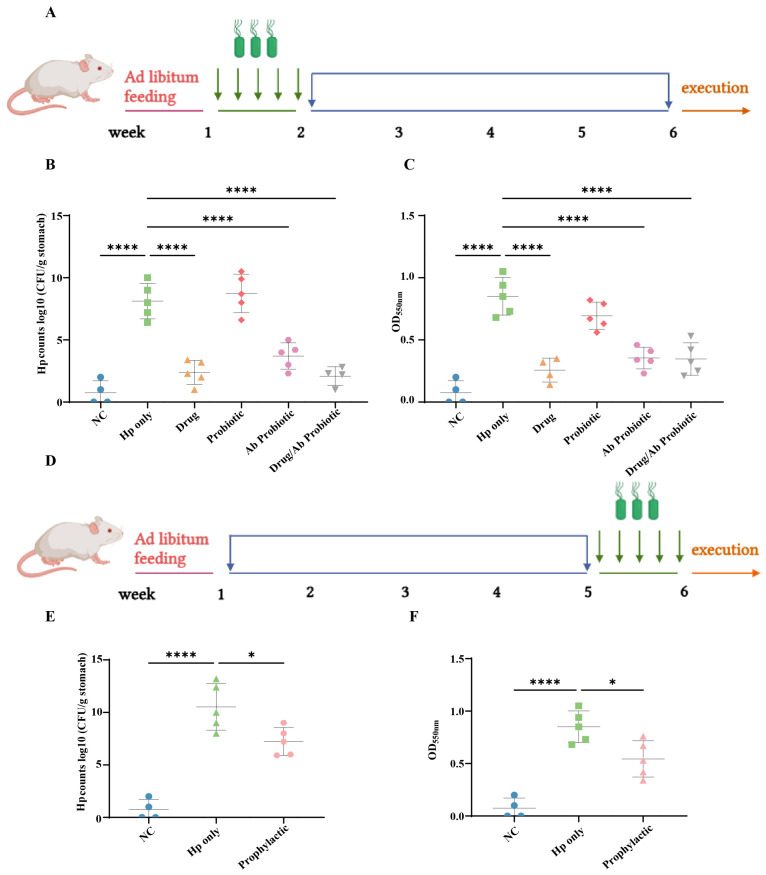
Mouse feeding regimens and assessment of gastric Hp colonization in treatment and prevention groups. (**A**) Mouse feeding regimens for the treatment group. Quantification of gastric *Helicobacter pylori* colonization in the treatment group using viable bacterial counts (**B**) and urea hydrolysis activity (phenol red assay) (**C**). (**D**) Mouse feeding regimens for the prevention group. Measurement of gastric *Helicobacter pylori* colonization in the prevention group through viable bacterial counts (**E**) and urea hydrolysis activity (phenol red assay) (**F**). * *p* < 0.05, **** *p* < 0.0001 compared to the Hp-only group.

To evaluate the anti-Hp efficacy of engineered probiotics, we established a murine infection model. Quantitative culture assays revealed significantly higher gastric Hp viable counts in the Hp-only group compared to the NC group (Figure 4B), confirming successful model establishment. While the Probiotic group showed no anti-Hp activity, the Drug, Ab Probiotic, and Drug/Ab Probiotic groups all showed significant reductions in Hp counts. The significant reduction in Hp levels in the treatment group indicated that both the active drug and engineered probiotics exhibited anti-Hp therapeutic effects, whereas wild-type probiotics failed to effectively treat Hp infection. In prophylactic studies, compared to the Hp-only group directly infected with Hp, the preventive group exhibited a significant reduction in Hp colonization, demonstrating that the engineered probiotics exerted a marked protective effect against Hp infection (Figure 4E).

Urease activity, quantified via phenol red indicator absorbance at 550 nm, correlated strongly with viable counts, further validating the therapeutic effects. Engineered probiotics significantly reduced Hp colonization in both therapeutic and prophylactic murine models, demonstrating potent anti-Hp efficacy, while non-antibody probiotics showed no effect (Figure 4C,F). These results indicated that the engineered probiotics exhibited effective therapeutic and prophylactic efficacy against Hp, significantly reducing gastric colonization and urease activity compared to ineffective wild-type probiotics.

### 3.5. Engineered Probiotics Regulate the Expression of Inflammatory Factors That Alleviate Hp Infection

Hp infection is closely associated with altered levels of inflammatory cytokines in the gastric mucosa, particularly TNF-α, IFN-γ, and IL-4 [27,28]. Studies have demonstrated that Hp infection upregulates IFN-γ and TNF-α expression in the gastric mucosa, with IFN-γ serving as a central mediator of the immune response. It not only induces apoptosis of gastric epithelial cells but also contributes to the chemogenic transformation of gastric mucosa [29]. Cytokine profiling in Hp-infected individuals reveals elevated levels of Th1-associated markers, including IFN-γ, IL-12, IL-18, and TNF-α, consistent with a predominant Th1-type immune response [30].

**Figure 5:**
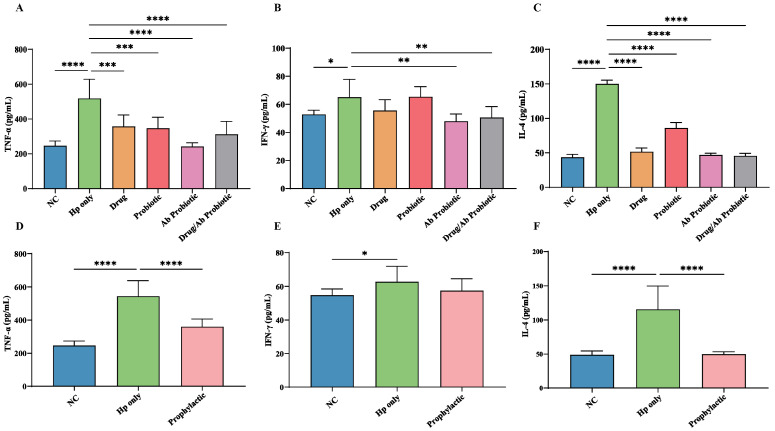
Cytokine levels in the serum of mice in treatment and prevention groups. Production amounts of TNF-α (**A**), IFN-γ (**B**), and IL-4 (**C**) in the serum of mice in the treatment group. Production amounts of TNF-α (**D**), IFN-γ (**E**), and IL-4 (**F**) in the serum of mice in the prevention group. * *p* < 0.05, ** *p* < 0.01, *** *p* < 0.001, **** *p* < 0.0001 compared to the Hp-only group.

Quantitative ELISA analysis of TNF-α, IFN-γ, and IL-4 (Figure 5A) showed that TNF-α production in the Hp-only group was substantially higher than in the Drug, Ab Probiotic, and Drug/Ab Probiotic groups, aligning with theoretical predictions. Notably, TNF-α levels in the Probiotic group were comparable to those in the Hp-only group, indicating that engineered probiotics effectively reduced TNF-α-mediated inflammation. While the Ab Probiotic group showed a limited effect on IFN-γ compared to the Hp-only group, the decrease was still evident (Figure 5B). Further analysis revealed that the Ab Probiotic group exhibited a marked reduction in IL-4 levels (Figure 5B,C).

In the prophylactic cohort, early administration of engineered probiotics significantly reduced TNF-α levels relative to the Hp-only group (Figure 5D), highlighting their potential for preventing Hp-induced inflammation. Although no clear effect on IFN-γ was observed (Figure 5E), engineered probiotics effectively downregulated IL-4 expression (Figure 5F). These results suggested that engineered probiotics effectively reduced Hp-associated gastric inflammation by significantly lowering TNF-α and IL-4 levels in both therapeutic and prophylactic settings, while non-antibody probiotics showed no anti-inflammatory benefits, with IFN-γ modulation remaining limited but supporting their role in balancing Th1/Th2 immune responses.

### 3.6. Engineered Probiotics Overinhibited Neutrophil Infiltration and Gastric Mucosal Structural Repair to Reduce Pathological Damage to Gastric Tissue Caused by Hp Infection

**Figure 6:**
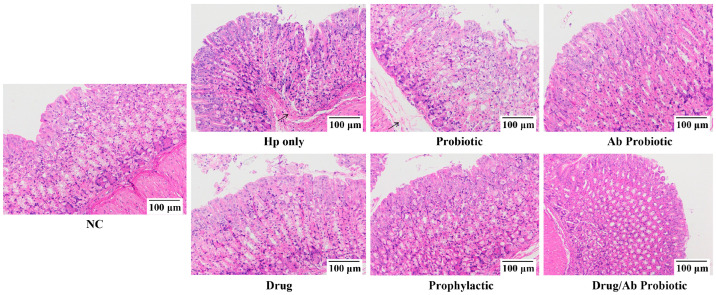
Histopathological analysis of gastric tissue sections in mice.

Gastric histopathological analysis provides critical insights into Hp-induced tissue damage severity. Histopathological examination of infected gastric tissue sections revealed characteristic pathological alterations, including lesion progression and structural deterioration. Both Hp-only and Probiotic groups exhibited pronounced neutrophil infiltration accompanied by severe mucosal damage, manifested through disorganized epithelial cell arrangement and loosened connective tissue architecture—histological hallmarks of Hp-driven inflammatory pathogenesis (Figure 6).

Therapeutic intervention with the Ab Probiotic group substantially alleviated these pathological changes. Compared to the Drug monotherapy group, the Drug/Ab Probiotic group demonstrated enhanced therapeutic efficacy, characterized by restored epithelial polarity and compact stromal organization. Notably, prophylactic administration in the Prophylactic group also attenuated histological damage compared to the Hp-only group, though to a lesser extent than therapeutic interventions (Figure 6). These findings establish that engineered probiotics mitigate Hp-induced gastropathy through two synergistic mechanisms: reducing neutrophil-mediated inflammatory infiltration and preserving the structural integrity of gastric mucosal epithelium and connective tissue matrix.

### 3.7. Engineered Probiotics Antagonize Hp Infection and Antibiotic Damage by Maintaining Gut Microbiota Diversity and Structural Remodeling

**Figure 7:**
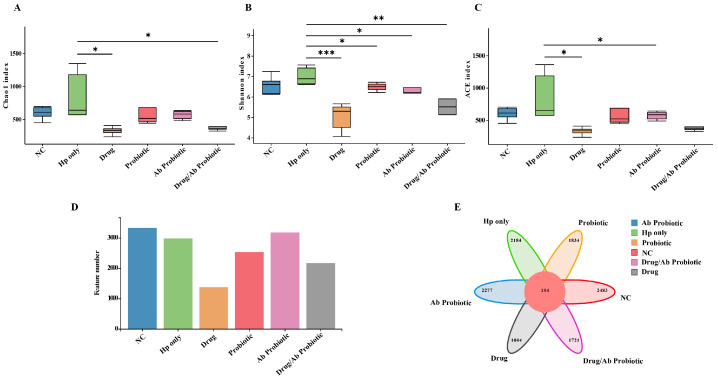
Intestinal diversity analysis of Alpha diversity index in the treatment group. The Chao1 and Ace indices measure species richness, that is, the number of species. The Shannon index is used to measure species diversity. The larger the Shannon index value, the higher the species diversity of the sample. (**A**) Chao1 index. (**B**) Shannon Index. (**C**) ACE index. (**D**) Distribution map of the number of OTUs in the treatment group. (**E**) Venn diagram, where each circle corresponds to a sample group. The overlapping regions represent the number of species shared between groups, while the non-overlapping areas indicate the unique species count specific to each individual group. * *p* < 0.05, ** *p* < 0.01, *** *p* < 0.001 compared to the Hp-only group.

16S rRNA sequencing serves as a critical methodology for evaluating gut microbiota dynamics during Hp infection and post-intervention phases. Analysis of intestinal alpha diversity indices in the treatment group included the Chao1 index (Figure 7A), Shannon index (Figure 7B), and ACE index (Figure 7C). The analysis showed significant statistical differences in Alpha diversity index in the treatment group of mice. Specifically, the Drug group of mice treated with antibiotics showed lower microbial diversity [31]. In contrast, the Ab Probiotic group mitigated this antibiotic-induced dysbiosis, demonstrating microbiota-preserving capacity. However, there was no significant difference in the Alpha diversity index between Hp-only and NC groups in the Ab Probiotic group and the control group, and no statistical difference in the Alpha diversity index between the same prevention groups.

**Figure 8:**
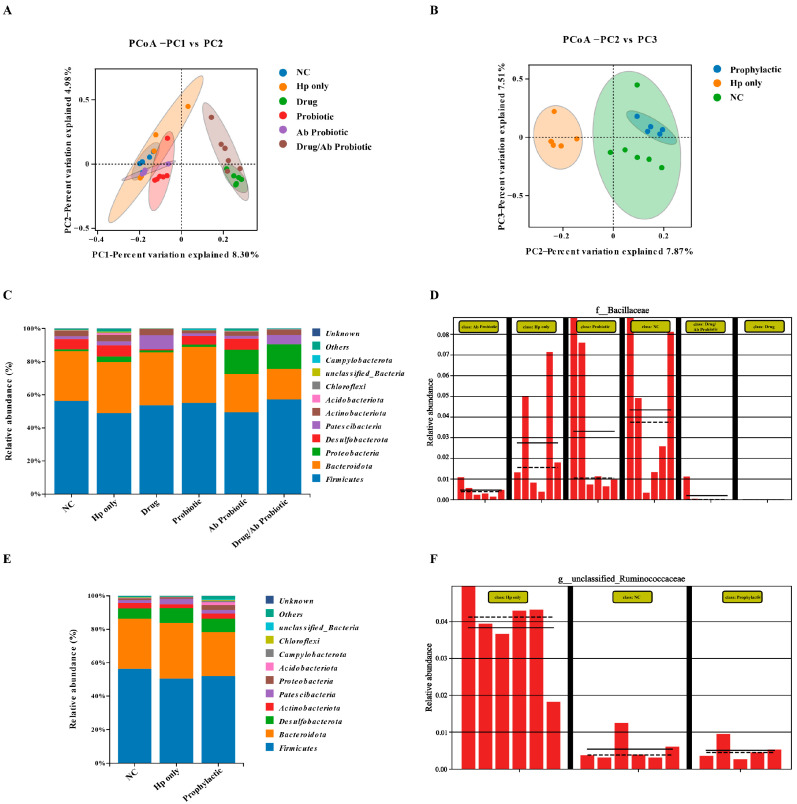
Effects of engineered probiotics on the gut microbiota of obese mice. PCoA in mice from the prevention group (**A**) and the treatment group (**B**) The horizontal and vertical coordinates represent the two characteristic values that cause the greatest differences among the samples, and the main degree of influence is reflected in the form of percentages. Comparison of species distribution between the treatment group (**C**) and the prevention group (**E**). Comparison of species abundance in the treatment group (**D**) and the prevention group (**F**).

Beta diversity analysis, as a core metric for assessing microbial community heterogeneity, effectively revealed structural differences through species composition quantification. This study utilized principal coordinate analysis (PCoA) and non-metric multidimensional scaling (NMDS) to visualize intergroup variations. Spatial distribution patterns demonstrated a significant negative correlation between sample distance and microbial similarity, closer proximity indicating higher homology in species composition and abundance characteristics. The distribution of OTU numbers in the treatment group (Figure 7D) and the Venn diagram (Figure 7E) revealed the proximity in the number of characteristics between the NC group and the Ab Probiotic group, which may reflect the positive impact of engineered probiotics on gut microbiota diversity. Notably, the Drug group exhibited severe microbiota disruption, contrasting with probiotic interventions that attenuated these detrimental effects. The PCoA results showed that the Hp-only group was significantly separated from the NC group in terms of β diversity, reflecting the success of the experimental modeling and the significant impact of Hp on the gut microbiota (Figure 8A,B). In addition, the intervention with engineered probiotics made the composition of the gut microbiota in the prevention and treatment groups more similar to that in the untreated NC group, while the two groups with positive drug intervention were significantly different from the NC group and deviated from the normal state. Therefore, it was inferred that the positive drug caused severe damage to the diversity of the gut microbiota, while the engineered probiotics made their gut composition closer to that of the normal group.

We analyzed the species distribution of the gut microbiota in different groups of mice (Figure 8C–E). Phylogenetic analysis revealed significant spatial heterogeneity in the gut microbiota of each group of mice at the phylum level. Based on the species annotation results, the top 10/15/20 species in each group in terms of maximum abundance at the phylum level were selected to generate a bar chart of relative species abundance. The results showed that the dominant phyla in the mouse gut were *Firmicutes*, *Bacteroidota*, and *Proteobacteria*, a distribution pattern that was evolutively conserved in the characteristics of the human gut microbiota. The distribution of species was more abundant in the Hp-only group after Hp infection, which was in line with the experimental conclusion of Lino et al. [32]. In the treatment group, Ab Probiotic, the number of Proteobacteria increased, which may be related to the EcN we added, and the species distribution was more abundant. It is speculated that the engineered probiotics increased some beneficial strain and thus alleviated Hp infection. In the abundance comparison (Figure 8D–F), the Hp-only abundance in the prevention group was relatively increased, while the species distribution in the Prophylactic group with early intervention of engineered probiotics was closer to that in the NC group. However, there was no significant difference between the NC group and the Hp-only group in the treatment group. These results demonstrated that compared to antibiotics, the engineered probiotic treatments preserved the microbiota’s diversity by restoring alpha diversity and modulating beta diversity, resulting in a microbial structure closer to that of the NC group. In contrast, the Drug group exhibited significant disruption of microbiota balance. Phylum-level analysis revealed that the intervention with engineered probiotics increased the abundance of Proteobacteria, thereby potentially modulating the overall structure of the gut microbiota or influencing host physiological processes associated with this bacterial phylum.

## 4. Discussion

This study employed AI-driven antibody optimization through AlphaFold2-based homology modeling of a fully humanized single-domain antibody, integrating three computational methods (mCSM-AB, I-Mutant, and FoldX) for design refinement. The approach enhanced antibody–antigen binding activity by 24.95%, achieving 65.6% binding efficiency compared to the original UreBAb variant, aligning with current trends in AI-augmented antibody engineering. As demonstrated by Alireza et al. computational optimization improved anti-CD20 nanobody (NB2) binding capacity by 14.4% [33], while Xin et al. achieved an 87.4-fold affinity enhancement for a CD47-targeting nanobody (M7), alongside improved thermal stability (+7.36 °C) [34]. These precedents validate the utility of computational strategies in enhancing both binding efficacy and structural stability. The I107W mutation, strategically positioned in the UreAb active pocket, formed a hydrogen bond with antigenic urease residue Y541. Recombinant expression and activity assays confirmed that substituting isoleucine with tryptophan at position 107 significantly improved Hp inhibition. This enhancement arises from strengthened hydrogen bonding and altered hydrophobic interactions mediated by tryptophan’s aromatic ring [35,36]. While isoleucine exhibits moderate hydrophobicity, tryptophan’s bulky side chain likely stabilizes urease binding through hydrophobic and π-stacking interactions [37]. We verified the feasibility of this method through molecular dynamics simulation. This mutation exemplifies AI’s capacity to streamline antibody optimization by predicting critical binding residues and screening mutants, thereby reducing experimental costs and development timelines.

Building on these computational advances, we engineered an EcN-based probiotic system for stable PTM151-I107WAb expression. In murine Hp infection models, the engineered probiotic significantly attenuated gastric inflammation and preserved mucosal integrity, contrasting with severe histopathological damage observed in both infected mice and positive drug-treated groups. These findings align with Miranda and Figueiredo’s [38] observations of infection-induced gastric lesions and Liu et al.’s correlation between Hp density and lesion severity [39]. Notably, our probiotic intervention mitigated these pathological effects, underscoring its therapeutic potential in maintaining gastric homeostasis.

Hp infection disrupts gut microbiota homeostasis by altering microbial relative abundance [40], likely attributed to its acid-neutralizing capacity. This environmental modulation facilitates the migration of acid-sensitive gastric microbes to the distal gastrointestinal tract, thereby perturbing gut microbiota composition [41,42]. Our engineered probiotic counteracts this dysbiosis through dual mechanisms: urease inhibition preserves gastric acidity, restricting abnormal translocation of acid-sensitive bacteria; immune modulation via microbiota stabilization [43,44]. Notably, gut–brain axis interactions may further contribute to therapeutic efficacy, as specific microbiota strains regulate stress responses and neurological functions [45]. Consistent with Lino et al.’s clinical findings [32], Hp infection increased gut microbial α-diversity, whereas antibiotic treatment drastically reduced it. In contrast, our engineered probiotic maintained microbial diversity indices comparable to those of the NC group, while the Drug group exhibited progressive divergence. Taxonomic analysis revealed *Firmicutes*, *Bacteroidetes*, and *Proteobacteria* as dominant phyla. The probiotic specifically enriched Proteobacteria abundance, restoring microbiota profiles in the prevention group to NC-like levels. These observations highlight a critical advantage over conventional antibiotic regimens, which indiscriminately eradicate commensal flora [46]. Comparative analysis of microbial relative abundance revealed distinct intervention outcomes between regimens. Therapeutic groups showed negligible divergence between NC and Hp-only groups, reflecting limited microbiota restoration during active Hp infection. In contrast, prophylactic intervention enhanced Hp-only subgroup abundance (indicating improved niche colonization) and achieved NC-like phylogenetic profiles in the Prophylactic group through preemptive Hp suppression, demonstrating superior ecological preservation. By targeting urease activity without broad-spectrum microbicidal effects, our engineered probiotic system offers a precision therapeutic alternative to mitigate antibiotic overuse and preserve ecological balance in gut microbiota.

While this study has demonstrated the anti-Hp potential of engineered probiotics, the field still faces two critical challenges that need to be addressed: the development of targeted delivery systems specifically for Hp gastric mucosal colonization and the comprehensive evaluation of long-term intervention effects on gastrointestinal microecological networks. Moreover, while this study advances AI-guided antibody optimization, certain limitations warrant discussion. Single-point mutagenesis may insufficiently drive comprehensive functional improvements, as structural cooperativity between residues often governs antibody performance. For instance, Chiba et al. achieved a 15 °C thermal stability enhancement in antibodies through synergistic double mutations (K43L/R109P) without compromising affinity [47]. Future work should prioritize deep learning-guided combinatorial mutagenesis to systematically explore structure–activity relationships across multiple residues. Additionally, the current evaluation framework focuses predominantly on binding activity and inflammatory markers. To fully characterize therapeutic potential, subsequent studies should incorporate multidimensional assessments of antibody neutralization efficacy, in vivo pharmacokinetics (e.g., half-life extension), and immunogenicity profiles [48]. Such refinements will bridge the gap between computational design and clinical translatability.

## 5. Conclusions

In summary, the study established an AI-guided probiotic system targeting Helicobacter pylori, featuring a rationally designed single-domain antibody mutant (I107W) with 65.6% urease inhibition. Engineered EcN expressing I107W significantly reduced Hp colonization and suppressed inflammation in mice, while preserving gut microbiota stability. This strategy outperformed conventional treatments, offering a precise, safe, and microecologically friendly approach. Future research should focus on clinical translation, and delivery optimization for targeted biotherapeutic applications.

## Figures and Tables

**Table 1:** Experimental protocol for evaluating the therapeutic and preventive effects of engineered probiotic EcN against Hp infection in mice.

Grouping	Processing Time (Days)	Method of Handling	Animal Numbers
NC	28	Physiological saline	10
Hp only	28	Physiological saline	16
Drug	28	Positive drug	10
Probiotic	28	Non-antibody EcN	10
Ab Probiotic	28	Antibody-engineered EcN	10
Drug/Ab Probiotic	28	Antibody-engineered EcN + Positive drug	10
Prophylactic	(Front) 28	Engineering EcN	10
	(after) 7	0.2 mol/L NaHCO_3_ + Hp	

Note: 200 μL of physiological saline was used daily. Positive drugs included potassium bicarbate tablets (12 mg/mL), tinidazole tablets (10 mg/mL), and clarithromycin tablets (5 mg/mL) dissolved in water according to the instructions; 200 μL of 10^9^ CFU engineered EcN gavage daily.

**Table 2:** Predicted single-point mutation sites and corresponding ΔΔG values for affinity optimization.

Mutation Site	mCMS-AB	Fold X	I-Mutant
K43L	0.100	−0.1298840	−0.02
K43T	0.056	−0.3313354	−0.97
K43M	0.019	−0.0915588	−0.25
E82W	0.124	−0.3929000	−0.03
I107P	0.083	−1.4463000	−2.33
I107W	0.178	−0.1029980	−1.78
I107F	0.146	−0.0543059	−1.03
R109P	0.033	−0.0887277	−0.85
R109N	0.007	−0.0101818	−0.03

## Data Availability

The data presented in this study are available within the article.

## Supplementary Materials

The following supporting information can be downloaded at: https://www.mdpi.com/article/10.3390/microorganisms13092043/s1, Table S1: Analysis of mutation sites; Table S2: Computational analysis of single−point mutations using mCSM−Ab; Table S3: Computational analysis of single−point mutations using FoldX; Table S4: Computational analysis of single−point mutations using i−mutant.

## Abbreviations

The following abbreviations are used in this manuscript:

Hp	*Helicobacter pylori*
EcN	*Escherichia coli* Nissle 1917
NC	Negative control
UreB	Urease subunit B
IARC	International Agency for Research on Cancer
NMDS	Non-metric multidimensional scaling
ANOVA	Analysis of variance
