# Supplementary material for: An AI-Designed Antibody-Engineered Probiotic Therapy Targeting Urease to Combat Helicobacter pylori Infection in Mice
Source: Microorganisms. 2025 Sep 1;13(9):2043. doi: 10.3390/microorganisms13092043 (PMC12473049; doi:10.3390/microorganisms13092043)
Supplement: Supplementary file 1 [file microorganisms-13-02043-s001.zip › microorganisms-3675359-supplementary.pdf]

Table S1 Analysis of mutation sites

InterProSurf	PyMol
S11	A14
S13	S15
K40	V16
P41	Q38
G42	Q39
K43	K40
P81	P41
E82	K43
F84	A44
T86	P45
K104	E82
E106	D83
I107	F84
K108	A85
R109	T86
A110	T103
A111	K104
-	I107
-	K108
-	R109

Table S2 Computational analysis of single-point mutations using mCSM-Ab

Residue	K40	P41	K43	E82	F84	T86	K104	I107	K108	R109
G	0.021	-0.041	0.108	-0.254	-0.632	-0.159	-0.199	-0.063	0.116	0.037
A	0.036	-0.034	0.107	-0.23	-0.611	-0.11	-0.163	-0.041	0.119	0.036
L	0.084	-0.012	0.1	-0.149	-0.504	0.04	-0.042	0.028	0.124	0.031
V	0.069	-0.019	0.103	-0.177	-0.547	-0.01	-0.083	0.004	0.123	0.033
I	0.084	-0.012	0.1	-0.149	-0.504	0.04	-0.042	\	0.124	0.031
P	0.069	\	0.103	-0.177	-0.547	-0.01	-0.083	0.004	0.123	0.033
R	-0.316	-0.256	-0.072	-0.243	-0.271	-0.534	-0.282	-0.139	-0.038	\
T	0.087	-0.116	0.056	-0.033	-0.417	\	-0.076	-0.036	0.096	0.024
S	0.062	-0.114	0.058	-0.062	-0.437	-0.485	-0.131	-0.047	0.092	0.024
C	-0.044	-0.226	-0.057	-0.056	-0.188	0.291	-0.04	-0.195	-0.064	-0.039
M	-0.08	-0.175	0.019	-0.203	-0.349	0.322	-0.117	-0.195	0.013	-0.002
K	\	-0.229	\	-0.047	-0.3	-0.254	\	-0.116	\	-0.014
E	0.097	-0.027	0.061	\	-0.098	0.406	0.053	0.002	0.077	0.009

Q	-0.079	-0.208	-0.016	-0.159	-0.45	-0.241	-0.199	-0.15	0.015	-0.022
D	0.074	-0.032	0.061	-0.041	-0.134	0.358	0.009	-0.014	0.072	0.009
N	-0.17	-0.19	0.009	-0.252	-0.53	-0.509	-0.34	-0.139	0.045	0.007
W	0.22	0.027	0.084	0.124	0.018	0.341	0.27	0.178	0.143	0.035
Y	0.165	0.026	0.112	0.009	-0.353	0.211	0.141	0.14	0.167	0.051
F	0.224	0.021	0.087	0.07	\	0.365	0.22	0.146	0.132	0.021
H	-0.154	-0.188	0.07	-0.229	-0.621	-0.128	-0.153	-0.148	0.105	0.093

Table S3 Computational analysis of single-point mutations using Fold X

Residue	K40	P41	K43	E82	F84	T86	K104	I107	K108	R109
G	1.58	1.42	1.05	0.06	3.76	1.91	1.63	1.54	0.30	0.27
A	1.13	1.10	0.78	0.22	3.29	0.68	1.12	0.83	0.15	0.24
L	0.18	1.18	-0.13	-0.48	0.69	-0.69	0.18	-0.23	0.10	-0.34
V	1.74	1.24	0.63	0.44	2.34	-0.66	0.42	0.44	1.61	0.16
I	1.15	1.24	0.11	0.14	1.26	-1.24	-0.17	\	1.21	-0.34
P	-1.65	\	4.83	-0.12	3.70	1.14	2.36	-1.14	7.07	-0.09
R	0.49	1.35	0.00	0.14	2.09	0.64	-0.43	0.09	-0.16	\
T	2.30	1.20	-0.33	0.67	2.05	\	0.74	0.09	1.33	-0.04
S	1.63	1.22	0.00	-0.20	2.85	1.37	1.12	0.44	0.84	0.10
C	1.46	1.33	0.51	0.21	2.50	0.62	0.77	0.93	0.31	0.21
M	0.38	0.95	-0.09	-0.54	0.93	-0.89	-0.08	-0.26	0.62	-0.38
K	\	1.11	\	-0.07	1.95	-0.49	\	0.17	\	-0.37
E	1.23	1.12	0.43	\	3.25	0.23	0.98	0.16	0.64	0.12
Q	1.32	1.20	0.21	-0.08	3.02	0.37	0.20	0.43	0.28	0.16
D	2.10	1.07	0.62	0.15	4.55	1.60	1.49	0.95	0.38	0.00
N	1.86	1.24	0.65	0.23	2.52	1.27	0.56	0.68	0.42	-0.01
W	2.98	1.17	0.26	-0.39	1.80	-0.32	1.25	-0.10	0.74	-0.58
Y	0.64	1.26	0.00	-0.11	-0.63	0.59	0.40	0.08	0.39	-0.66
F	0.57	1.17	-0.06	-0.21	\	-0.60	0.02	-0.05	0.36	-0.69
H	2.00	1.24	0.42	0.29	1.83	1.62	0.78	0.95	0.40	0.09

Table S4 Computational analysis of single-point mutations using i-mutant

Residue	K40	P41	K43	E82	F84	T86	K104	I107	K108	R109
G	-0.39	-1.11	-0.17	-1.44	-3.79	-1.78	-0.92	-3.13	-0.62	-0.31
A	-0.34	-1.76	-0.19	-1.16	-2.57	-0.12	-0.21	-1.91	-0.06	-0.11
L	-0.29	-1.93	-0.02	0.61	-1.67	0.39	1.33	1.22	1.24	0.7
V	0.68	-0.97	0.66	0.44	-2.68	0.16	0.68	-0.35	0.63	-0.04
I	0.55	-0.54	0.48	1.15	-0.52	0.6	0.48		0.5	0.45
P	0.5		0.39	-0.21	-1.54	-0.31	0.43	-2.33	0.2	-0.85
R	-0.21	-0.64	-0.14	-0.25	-1.72	0.92	-0.69	-1.51	-0.39	
T	-0.79	-1.88	-0.97	-0.57	-2.24		-0.2	-1.46	-0.05	0.28
S	-0.58	-1.96	-0.67	-0.87	-2.56	-0.13	-0.11	-1.71	0.13	-1.04
C	-0.31	-1.77	-0.27	-0.42	-1.62	0.29	0.57	-2.05	0.41	0.07
M	-0.33	-1.21	-0.25	0.27	-0.87	0.8	0.08	-0.16	0.35	-0.07

K		-1.4		-1.19	-1.67	-0.32		-1.87		-0.49
E	0.04	-0.8	-0.05		-1.38	0.56	0.02	-0.88	-0.12	0.46
Q	-0.24	-1.17	0	-0.34	-1.86	-0.52	-0.28	-1.14	-0.09	0.17
D	-0.16	-1.49	-0.89	-0.25	-2.31	0.75	-0.38	-1.97	-0.76	-0.43
N	-0.57	-1.58	-0.06	-0.89	-1.74	0.29	-0.24	0.32	0.42	-0.03
W	-0.3	-1.39	-0.72	-0.03	-0.21	1.13	-0.33	-1.78	-0.4	0.03
Y	0.62	-1.32	0.53	0.43	-0.18	2.15	0.65	-1.68	0.78	0.43
F	1	-0.65	0.66	0.7		0.88	0.78	-1.03	1.17	0.37
H	-0.37	-1.57	-0.51	-0.45	-2.14	-0.33	0.1	-1.41	-0.12	-0.05
